# Extensively drug-resistant *Acinetobacter baumannii* in a Thai hospital: a molecular epidemiologic analysis and identification of bactericidal Polymyxin B-based combinations

**DOI:** 10.1186/s13756-015-0043-x

**Published:** 2015-01-29

**Authors:** Jocelyn Teo, Tze-Peng Lim, Li-Yang Hsu, Thean-Yen Tan, Suranthran Sasikala, Pei-Yun Hon, Andrea L Kwa, Anucha Apisarnthanarak

**Affiliations:** Department of Pharmacy, Singapore General Hospital, Outram Road, Singapore, 169608 Singapore; Division of Infectious Diseases, Department of Medicine, 1E Kent Ridge Road, NUHS, Yong Loo Lin School of Medicine, National University of Singapore, Tower Block, Level 9, Singapore, 119228 Singapore; Department of Laboratory Medicine, Changi General Hospital, 2 Simei St 3, Singapore, 529889 Singapore; Emerging Infectious Diseases, Duke-NUS Graduate Medical School, Singapore, 169857 Singapore; Division of Infectious Diseases, Faculty of Medicine, Thammasat University Hospital, Pathumthani, 12120 Thailand; Current address: A*Star, Biopolis, 31 Biopolis Way, Singapore, 138668 Singapore; Current address: Tan Tock Seng Hospital, 11 Jalan Tan Tock Seng, Singapore, 308433 Singapore

**Keywords:** Combination therapy, Carbapenem resistance, *Acinetobacter baumannii*

## Abstract

**Background:**

Limited knowledge of the local molecular epidemiology and the paucity of new effective antibiotics has resulted in an immense challenge in the control and treatment of extensively drug-resistant (XDR) *Acinetobacter baumannii* infections in Thailand. Antimicrobial combination regimens may be the only feasible treatment option in such cases. We sought to characterize the local molecular epidemiology and assess the bactericidal activity of various antibiotics individually and in combination against XDR *A. baumannii* in a Thai hospital*.*

**Methods:**

All XDR *A. baumannii* isolates from Thammasat University Hospital were collected between October 2010 and May 2011. Susceptibility testing was conducted according to reference broth dilution methods. Pulse-field gel electrophoresis was used to genotype the isolates. Carbapenemase genes were detected using polymerase chain reaction. *In vitro* testing of clinically-relevant concentrations of imipenem, meropenem, doripenem, rifampicin and tigecycline alone and in combination with polymyxin B was conducted using multiple combination bactericidal testing.

**Results:**

Forty-nine polymyxin B-susceptible XDR *A. baumannii* isolates were identified. *bla*_OXA-23_ and *bla*_OXA-51_ genes were detected in all isolates. Eight clonally related clusters were identified, resulting in the initiation of several infection control measures. Imipenem, meropenem, doripenem, rifampicin, and tigecycline in combination with PB respectively, exhibited bactericidal killing in 100%, 100%, 98.0%, 100% and 87.8% isolates respectively at 24 hours.

**Conclusion:**

Molecular epidemiologic analysis can aid the early detection of infection outbreak within the institution, resulting in the rapid containment of the outbreak. Imipenem/meropenem/rifampicin in combination with polymyxin B demonstrated consistent bactericidal effect against 49 *bla*_OXA-23_-harbouring XDR *A. baumannii* clinical isolates, suggesting a role of combination therapy in the treatment of these infections.

## Background

*Acinetobacter baumannii* (AB) is one of the leading causes of various nosocomial infections. Its success is due to its resilient and virulent properties, as well as its potential to acquire a plethora of drug resistance mechanisms [1]. In Thailand, AB infections represent a key healthcare issue – national surveillance detected a dramatic increase in carbapenem-resistant AB from 2.1% in 2000 to 46.7% in 2005 [2].

The management of these extensively drug-resistant (XDR) AB infections is particularly challenging due to the limited molecular epidemiologic data available. The drying antibiotic pipeline has also put us at risk of having no effective treatment options against infections caused by this bacterium in the near future [3]. This dearth in new antibiotics has led to the renewed clinical use of polymyxin antibiotics, the last resort treatment option for XDR AB infections [4]. However, there are few concerns related to polymyxin monotherapy, the primary being the emergence of heteroresistance and adaptive resistance, potentially leading to clinical failure [5-7].

As a consequence of this seemingly futile situation, combination antibiotic therapies are explored as potential therapeutic options. Previous *in vitro* studies investigating the benefits of combination therapy for the treatment of XDR AB suggest that effective combinations may be strain- or clone-specific [8-10]. As limited combination studies were performed on local Thai isolates [11-14], the objective of this study was to assess the bactericidal activity of various antibiotics individually and in combination against XDR AB, in addition to the characterization of the molecular epidemiology of XDR AB in Thailand*.*

## Materials and methods

### Microorganisms

Forty-nine clinical AB isolates from various sites (body fluid, blood, pus, sputum and urine) were collected from Thammasat University Hospital between October 2010 and May 2011. The isolates were identified via a commercial biochemical identification system using colonies obtained from overnight growth on solid media (API 20NE) (bioMérieux, Marcy l’Etoile, France). The bacteria were stored at −70°C in Protect® storage vials (Key Scientific Products, Inc., Stamford, TX, USA). Fresh isolates were sub-cultured twice on 5% blood agar plates (Thermo Scientific Microbiology, Malaysia) for 24 hours at 35°C prior to each experiment.

### Genotypic identification

Clonal relatedness was determined by pulse-field gel electrophoresis (PFGE). PFGE was performed on chromosomal DNA extracts after digestion using *Apa*I [15]. Digital images of the DNA fingerprints were processed using Molecular Analyst v1.6 (Bio-Rad, Hercules, CA, USA) and analyzed using the Dice coefficient and the unweighted-pair group method using average linkages. Isolates with similarity coefficients greater than 90% were considered to be within the same clonal cluster.

### Resistance gene testing

Screening for the presence of genes encoding OXA-type carbapenemases (*bla*_OXA-23_-like, *bla*_OXA-24_-like, *bla*_OXA-58_-like, and *bla*_OXA-51_-like genes) and metallo-beta-lactamases (MBL) (*bla*_IMP_, *bla*_VIM_, *bla*_GIM_, *bla*_SIM_, *bla*_SPM_) was performed via multiplex polymerase chain reactions (PCR) using protocols previously described in literature [16,17].

### Minimum inhibitory concentration testing

Minimum inhibitory concentrations (MIC) to imipenem, meropenem, doripenem, rifampicin, tigecycline and polymyxin B (PB) were obtained using broth dilution methods in accordance to Clinical and Laboratory Standards (CLSI) guidelines [18]. Categorical susceptibility was based on CLSI guidelines except for rifampicin and tigecycline, for which there are no standard breakpoints [19]. Breakpoints for tigecycline susceptibility were interpreted according to the US Food and Drug Administration standards for *Enterobacteriaceae spp*.

### Antimicrobial agents

Meropenem was obtained from AstraZeneca Inc. Imipenem was obtained from Merck Sharp & Dohme (I.A.) Corp. Doripenem was obtained from Johnson & Johnson Pharmaceutical Research and Development. PB and rifampicin were obtained from Sigma-Aldrich (St Louis, MO, USA). Tigecycline was obtained from Pfizer Inc.

### Multiple combination bactericidal testing (MCBT)

MCBT was conducted with imipenem, meropenem, doripenem, rifampicin and tigecycline alone and in combination with PB. Clinically-relevant (achievable) unbound concentrations of each antibiotic in blood or tissues were used. Hence, the meropenem concentration was 64 mg/L, representing a steady-state free peak concentration arising from a 2 g, 3-hour infusion dose [20]. Imipenem concentration was 32 mg/L, which simulates the steady-state free peak concentration arising from a 1 g, 40-min infusion [21]. Doripenem concentration was 13 mg/L, which corresponds to the steady-state free peak concentration arising from a 1 g, 4-hour infusion [22]. The simulated steady-state drug concentrations were 2 mg/L (free or unbound protein fraction) for PB, rifampicin and tigecycline, which corresponds to levels achieved with maximum intravenous doses of at least 1 million units of PB, 600 mg of rifampicin and 100 mg of tigecycline respectively [23-25].

The experiment was conducted using 96-well, round-bottom microtitre trays (Greiner Bio-One, Frickenhausen, Germany). Working antibiotic solutions were prepared at four times the required concentration. Each of one or two antibiotics was added in 50 μL aliquots. Wells containing only one antibiotic were topped up with 50 μL of sterile water, such that all wells had a volume of 100 uL before the addition of the bacterial suspension. Growth and sterility wells were included in each tray. The trays were then stored at −70°C and thawed before the start of each experiment.

Overnight bacterial cultures were diluted with pre-warmed cation-adjusted Mueller-Hinton broth (Ca-MHB) (BBL, BD Diagnostic Systems, Sparks, MD, USA) and incubated further at 35°C until log-phase growth was reached. The bacterial inoculum was prepared by dilution with Ca-MHB according to an ultraviolet spectrophotometry calibration method (absorbance at 630 nm); 100 μL of the suspension was transferred to each well, resulting in a final inoculum approximating 1 × 10^5^ – 5 × 10^5^ CFU/mL and the desired concentration of antimicrobials in each well. Each MCBT tray was then incubated in a humidified incubator (35°C) for 24 hours.

The plates were examined for turbidity at 24 hours and entire contents of each well (200 μL) were obtained for quantitative culture. Samples were centrifuged at 10,000 × *g* for 15 minutes followed by decanting. The pellets were then reconstituted with sterile normal saline to their original volumes in order to minimize drug carryover effect. Total bacterial populations were quantified by spiral plating 10× serial dilutions of the samples (50 μL) onto Mueller-Hinton agar plates. The plates were incubated in a humidified incubator for up to 24 hours and the bacterial density from each sample was enumerated visually. The theoretical reliable lower limit of detection was 400 CFU/mL.

Bactericidal activity was defined as a ≥ 3 log_10_ CFU/mL decrease in the colony count from the initial inoculum at 24 hours, as the primary endpoint. This endpoint is synonymous with sustained killing effect of ≥ 99.9% [26].

### Emergence of resistance studies

To analyze the potential presence of PB heteroresistance, 24-hour PB time-kill studies were conducted as previously described [9]. Briefly, colonies recovered at 24 hours in the time-kill studies were cultured on Mueller-Hinton agar supplemented with PB at 3 times the initial MIC of PB. Repeat MIC testing was then performed on three randomly chosen colonies recovered 24 hours post-PB exposure on the PB-containing plates. Heteroresistance was defined by growth of colonies on PB-containing plates, with confirmation of an increased MIC of more than three times the initial MIC upon repeat testing.

## Results

### Susceptibility studies

**Figure 1:**
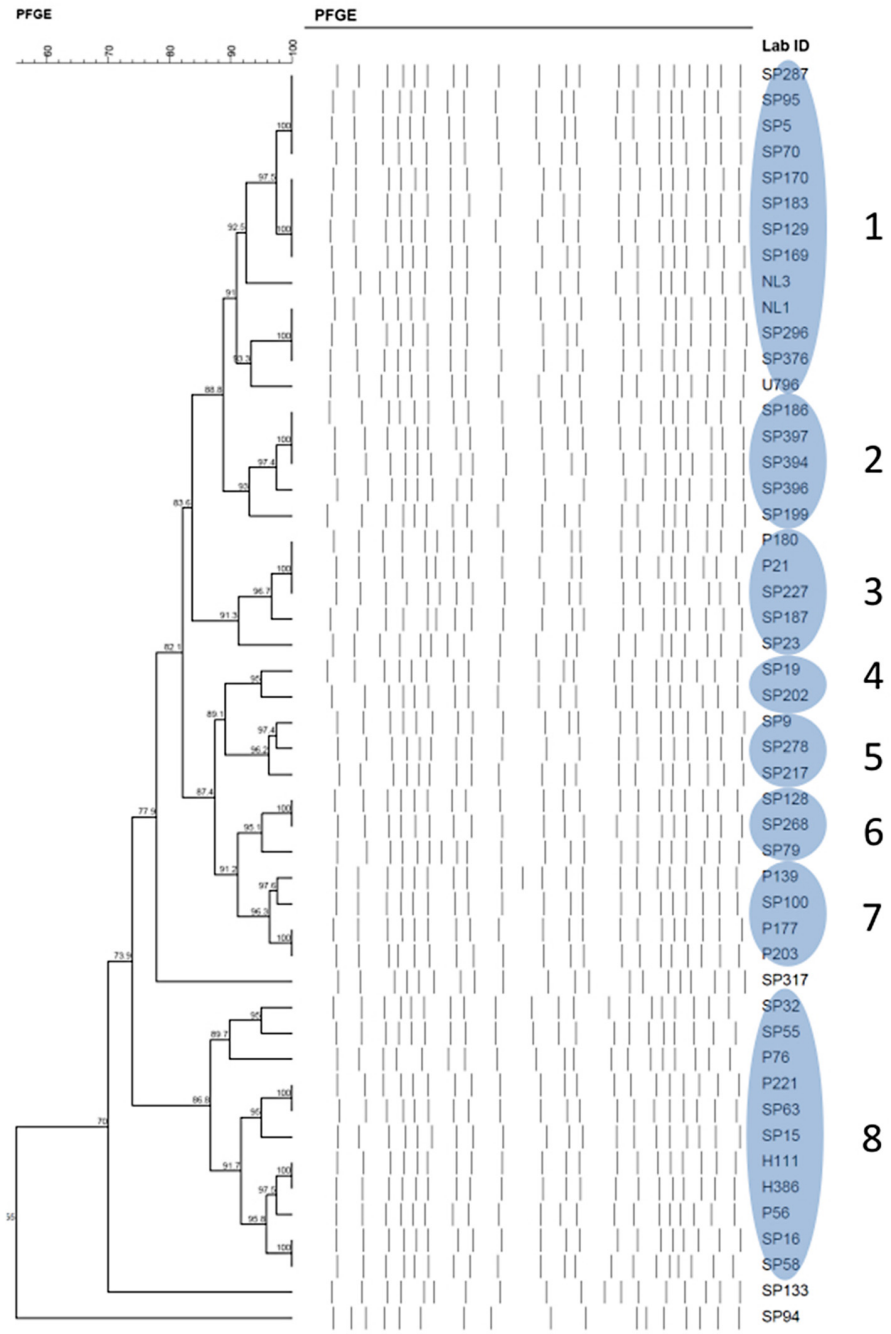
**Phylogenetic tree diagram.** A blue oval shape denotes a clonal group after applying a similarity index of ≥ 90%.

Forty-nine XDR AB isolates were identified. All isolates were resistant to meropenem, imipenem, ampicillin/sulbactam, ciprofloxacin, gentamicin, aztreonam, piperacillin/tazobactam, ceftazidime, cefepime and amikacin based on susceptibility testing results from Thailand (data not shown). They remained susceptible to PB (MIC range 0.5–2 mg/L). The MICs of rifampicin and tigecycline ranged from 4–16 mg/L and 1–16 mg/L respectively (Figure 1).

### Clonal relationship analysis

**Figure 2:**
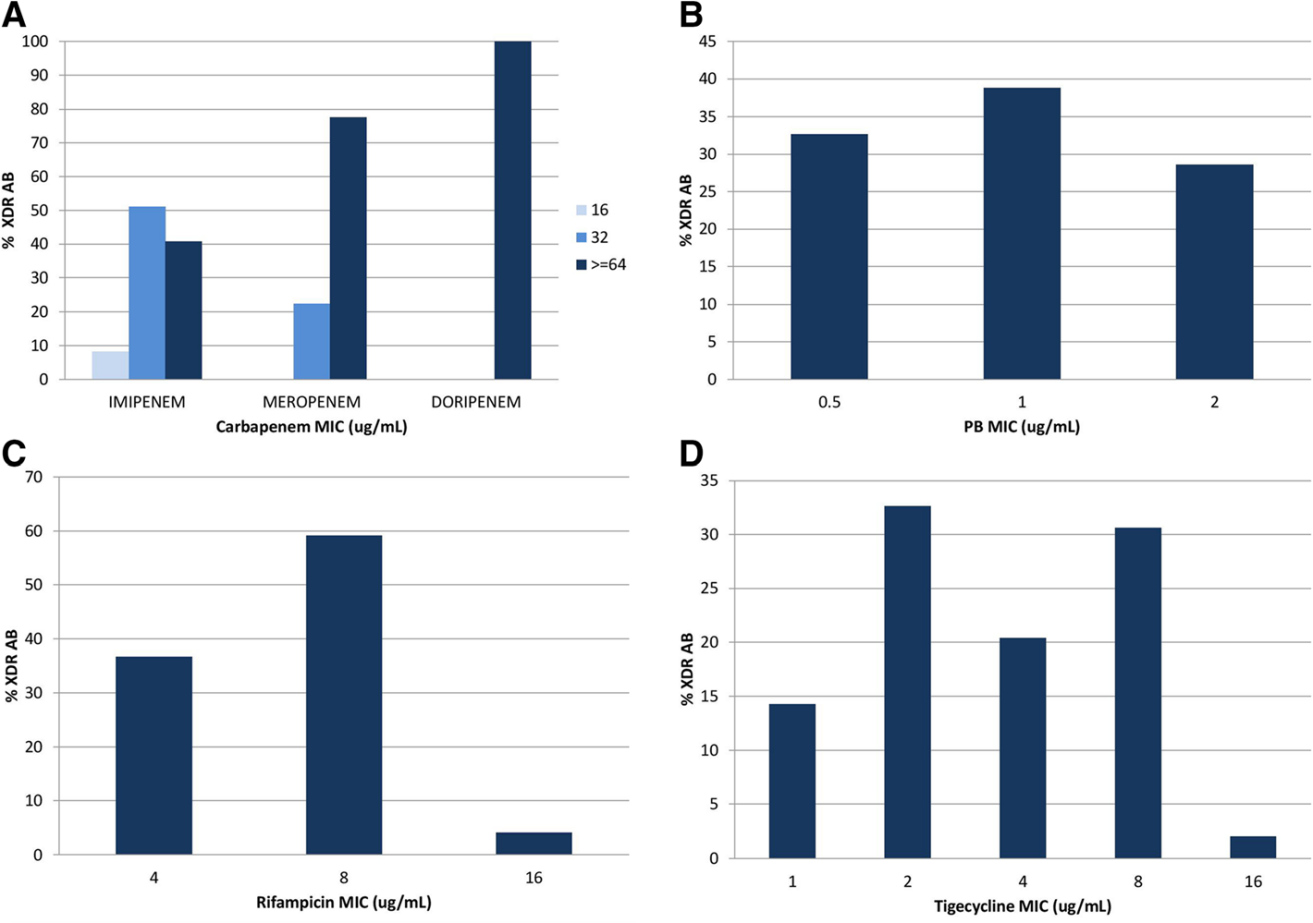
**Susceptibilities of XDR AB to carbapenems (A), polymyxin B (B), rifampicin (C) and tigecycline (D).**

The phylogenetic dendrogram of the XDR AB isolates is shown in Figure 2. Eight clonal clusters containing 46 isolates were identified. The remaining three isolates were clonally unrelated. Majority of the isolates (37/49, 75.5%) were retrieved from the medical units. Interestingly, the five isolates in clonal cluster 3 were retrieved from the same surgical intensive care unit. During the study period, real-time feedback of molecular epidemiologic results was performed, followed by immediate implementation of infection control measures, leading to the reduction in transmission of XDR AB within the institution [27,28].

### Presence of resistance genes

*bla*_OXA-51_ gene, a naturally occurring resistance gene unique to AB, was detected in all study isolates. In addition, it was observed that all isolates were *bla*_OXA-23_ -harbouring, a phenomenon reported to be highly-prevalent in Thailand hospitals [29,30]. MBL genes were absent in our study isolates.

### MCBT studies

In single antibiotic MCBT, none of the antibiotics exhibited bactericidal activity against all isolates at 24 hours except for PB and tigecycline. Tigecycline was bactericidal against 14 out of 49 isolates (28.6%) while PB was bactericidal against 43 out of 49 isolates (87.8%) (Table 1).

**Table 1:** **Bactericidal activity of single antibiotics and combination antibiotics against XDR AB**

**Single antibiotic**	**Bactericidal against XDR AB (%)**
Imipenem	0
Meropenem	0
Doripenem	0
PB	87.8
Rifampicin	0
Tigecycline	28.6
**Combination antibiotics**	
Imipenem-PB	100
Meropenem-PB	100
Doripenem-PB	98.0
Rifampicin-PB	100
Tigecycline-PB	87.8

In combination MCBT, imipenem-PB, meropenem-PB and rifampicin-PB combinations were bactericidal against all 49 isolates, including the six isolates where PB alone was not bactericidal. Doripenem-PB was not bactericidal against only one isolate. Tigecycline-PB was only bactericidal against 87.8% of the isolates. Tigecycline MICs of isolates where the tigecycline-PB combination failed were ≥ 2 mg/L.

### Emergence of resistance studies

PB alone was found to be rapidly bactericidal at 6 h before regrowth occurred at 24 h with a final inoculum of 3.40 to 6.50 log CFU/mL. Colonies were isolated on PB-containing plates for four isolates. Repeat MIC testing of three randomly chosen colonies from these four isolates demonstrated a significant elevation in PB MIC (256 mg/L). This suggests that PB heteroresistance may be present in this group of XDR AB isolates.

## Discussion

There were some key findings in this study. Firstly, nearly all XDR AB isolates were clonally related and had the same resistance gene profile. Secondly, our results showed that PB in combination with either imipenem, meropenem or rifampicin was consistently bactericidal for all of our XDR AB isolates. The high clonal relatedness suggests that creation of unit-specific antibiograms and combination antibiograms based on our findings may have a potential role in the guidance of appropriate empirical combination therapy.

Our study suggested the feasibility of using molecular epidemiologic analysis to limit the transmission of XDR AB in a resource-limited setting in Thailand. At the beginning of our study (October 2010), the hospital saw an increase in the incidence of XDR AB. Molecular analysis (including resistance gene testing), which was continued throughout our study period, identified that the isolates were clonally related. This information was pivotal in identifying the outbreak. At the time of the study, standard infection control measures such as hand hygiene and contact isolation precautions, as well as an antimicrobial stewardship programme were already present. In response to the molecular results, new measures including environmental cleaning, active surveillance in index units and cohorting were promptly introduced by December 2010. Advanced source control (chlorhexidine bath and oral care) was also implemented in intensive care units. In addition, staff education programmes were organised and adherence monitoring with unit-specific feedback was introduced. Following the implementation, incidence of XDR AB slowly decreased [27,28]. Although molecular epidemiologic analysis was not continued after the study period, data from our study highlight the utility of such a strategy in outbreak control.

We noted that *bla*_OXA-23_ was a major resistance determinant in our study. This observation concurred with studies in the Southeast Asian and Asian region. *bla*_OXA-23_ genes were detected in most of the carbapenemase-producing AB (91%) in a Singapore hospital, while *bla*_IMP-4_ and *bla*_OXA-58_ genes were also detected in a few isolates [15]. *bla*_OXA-23_ is also highly prevalent in countries such as China and Korea, where 40 – 100% of the carbapenem-resistant AB isolates were found to carry the gene [31,32].

In our study, we also observed that antimicrobial monotherapies had little or no bactericidal activity, with the exception of PB which retained bactericidal activity in the majority of the isolates. However, when PB monotherapy is employed, there is the possibility of the emergence of heteroresistant populations [6]. The inability of PB monotherapy to be universally bactericidal, despite all isolates having MICs ≤ 2 mg/L, suggested the presence of such heteroresistant populations in our isolates. Indeed, further testing proved that a number of isolates exhibited increased MIC post-PB exposure in our study. This underscores the need for combination therapies to reduce the chance of clinical failure. The high *in vitro* bactericidal activity (near 100% of all combinations except tigecycline-PB) of the combinations suggested that combination therapy may overcome the selection of PB-heteroresistant populations associated with PB monotherapy. Hence, one potential interesting approach to aid the reduction of antibiotic resistance is to use unit-specific combination antibiograms to help guide empirical therapy. The use of combination antibiograms had been shown to help clinicians provide early appropriate antimicrobial therapy in an XDR AB endemic setting [33]. *In vitro* and animal studies have suggested promising synergism/bactericidal activity of antibiotic combinations against highly-resistant AB [8-10,34]. A recent systematic review has also elucidated that carbapenem-polymyxin combinations have been associated with high *in vitro* synergism, low antagonism and reduced development of resistance especially for resistant AB infections [35]. Likewise, we demonstrated that combination therapy, PB in combination with an anti-pseudomonal carbapenem or rifampicin in our case, exhibited bactericidal activity *in vitro* against XDR AB.

The utility of combination therapy has also been evaluated in the clinical setting. Whilst the limited randomized controlled trials available were unable to conclusively demonstrate the clinical benefit of combination therapy in the clinical setting [36,37], results from observational studies have suggested that combination therapy may be beneficial for the treatment of carbapenem-resistant AB in Thailand [38,39] and elsewhere [40].

We had elected to perform our *in vitro* tests using MCBT [41,42]. While MCBT has not been used in the region, variations of this testing method have been adopted elsewhere to investigate combination therapies [43,44]. Our method allowed the efficient measurement of bactericidal activity of multiple different combinations against XDR AB at any one time in a 96-well plate. Furthermore, maximal clinically-achievable unbound concentrations of antibiotics were employed to mimic the *in vivo* killing effect when maximum antibiotic doses were used, allowing better clinical extrapolation of the results.

Our study is limited by the nature of study isolates from a single institution and the assessment of PB-susceptible strains which may not allow the generalizability of our results. In addition, *in vitro* synergism and/or bactericidal activity are highly dependent on the test methods, of which currently has not been standardized. It is unknown which method best predicts *in vivo* efficacy. It appears that our test method overestimates the bactericidal activity of polymyxin monotherapy. The heteroresistance phenomenon was not detected in our MCBT experiments. One of the reasons could be due to the small test volumes utilized in our experiments, and the corresponding likelihood that the percentage of hetero-resistant sub-population is very minute. As a result, it is difficult to elucidate such strains through small quantitative cultures. Furthermore, until resources are readily available, we are unable to implement MCBT as a routine test in our clinical setting.

## Conclusions

The lack of therapeutic options in the treatment of XDR AB infections and risk of clinical failure associated with polymyxin monotherapy highlighted the need to identify effective combination therapies. The clinical relevance of molecular epidemiology in the containment of XDR AB infections together with the effectiveness of combination therapy should be further studied in a resource-limited setting.

## Abbreviations

AB: *Acinetobacter baumannii*
MIC: Minimum inhibitory concentration
MCBT: Multiple combination bactericidal testing
MBL: Metallo-beta-lactamase
PCR: Polymerase chain reaction
PB: Polymyxin B
PFGE: Pulse-field gel electrophoresis
XDR: Extensively drug-resistant

## References

[1] Peleg AY, Seifert H, Paterson DL (2008). Acinetobacter baumannii: emergence of a successful pathogen. Clin Microbiol Rev.

[2] Apisarnthanarak A, Buppunharun W, Tiengrim S, Sawanpanyalert P, Aswapokee N (2009). An overview of antimicrobial susceptibility patterns for gram-negative bacteria from the National Antimicrobial Resistance Surveillance Thailand (NARST) program from 2000 to 2005. J Med Assoc Thai.

[3] Boucher HW, Talbot GH, Bradley JS, Edwards JE, Gilbert D, Rice LB, Scheld M, Spellberg B, Bartlett J (2009). Bad bugs, no drugs: no ESKAPE! An update from the Infectious Diseases Society of America. Clin Infect Dis.

[4] Zavascki AP, Goldani LZ, Li J, Nation RL (2007). Polymyxin B for the treatment of multidrug-resistant pathogens: a critical review. J Antimicrob Chemother.

[5] Barin J, Martins AF, Heineck BL, Barth AL, Zavascki AP (2013). Hetero- and adaptive resistance to polymyxin B in OXA-23-producing carbapenem-resistant Acinetobacter baumannii isolates. Ann Clin Microbiol Antimicrob.

[6] Li J, Rayner CR, Nation RL, Owen RJ, Spelman D, Tan KE, Liolios L (2006). Heteroresistance to colistin in multidrug-resistant Acinetobacter baumannii. Antimicrob Agents Chemother.

[7] Cai Y, Chai D, Wang R, Liang B, Bai N (2012). Colistin resistance of Acinetobacter baumannii: clinical reports, mechanisms and antimicrobial strategies. J Antimicrob Chemother.

[8] Yoon J, Urban C, Terzian C, Mariano N, Rahal JJ (2004). In vitro double and triple synergistic activities of Polymyxin B, imipenem, and rifampin against multidrug-resistant Acinetobacter baumannii. Antimicrob Agents Chemother.

[9] Lim TP, Tan TY, Lee W, Sasikala S, Tan TT, Hsu LY, Kwa AL (2011). In-vitro activity of polymyxin B, rifampicin, tigecycline alone and in combination against carbapenem-resistant Acinetobacter baumannii in Singapore. PLoS One.

[10] Lim TP, Tan TY, Lee W, Sasikala S, Tan TT, Hsu LY, Kwa AL (2009). In vitro activity of various combinations of antimicrobials against carbapenem-resistant Acinetobacter species in Singapore. J Antibiot (Tokyo).

[11] Santimaleeworagun W, Wongpoowarak P, Chayakul P, Pattharachayakul S, Tansakul P, Garey KW (2011). In vitro activity of colistin or sulbactam in combination with fosfomycin or imipenem against clinical isolates of carbapenem-resistant Acinetobacter baumannii producing OXA-23 carbapenemases. Southeast Asian J Trop Med Public Health.

[12] Kiratisin P, Apisarnthanarak A, Kaewdaeng S (2010). Synergistic activities between carbapenems and other antimicrobial agents against Acinetobacter baumannii including multidrug-resistant and extensively drug-resistant isolates. Int J Antimicrob Agents.

[13] Pongpech P, Amornnopparattanakul S, Panapakdee S, Fungwithaya S, Nannha P, Dhiraputra C, Leelarasamee A (2010). Antibacterial activity of carbapenem-based combinations againts multidrug-resistant Acinetobacter baumannii. J Med Assoc Thai.

[14] Thamlikitkul V, Tiengrim S (2014). In vitro activity of colistin plus sulbactam against extensive-drug-resistant Acinetobacter baumannii by checkerboard method. J Med Assoc Thai.

[15] Koh TH, Sng LH, Wang GC, Hsu LY, Zhao Y (2007). IMP-4 and OXA beta-lactamases in Acinetobacter baumannii from Singapore. J Antimicrob Chemother.

[16] Ellington MJ, Kistler J, Livermore DM, Woodford N (2007). Multiplex PCR for rapid detection of genes encoding acquired metallo-beta-lactamases. J Antimicrob Chemother.

[17] Woodford N, Ellington MJ, Coelho JM, Turton JF, Ward ME, Brown S, Amyes SG, Livermore DM (2006). Multiplex PCR for genes encoding prevalent OXA carbapenemases in Acinetobacter spp. Int J Antimicrob Agents.

[18] Clinical and Laboratory Standards Institute. Methods for Dilution: Antimicrobial Susceptibility Tests for Bacteria That Grow Aerobically; Approved Standard - Eighth Edition M07-A9. CLSI. Wayne, Pennsylvania, USA;2009.

[19] Clinical and Laboratory Standards Institute. Performance standards for antimicrobial testing: Seventeenth Informational Supplement M100-S20. CLSI. Wayne, Pennsylvania, USA;2010.

[20] Jaruratanasirikul S, Sriwiriyajan S, Punyo J (2005). Comparison of the pharmacodynamics of meropenem in patients with ventilator-associated pneumonia following administration by 3-hour infusion or bolus injection. Antimicrob Agents Chemother.

[21] Sakka SG, Glauner AK, Bulitta JB, Kinzig-Schippers M, Pfister W, Drusano GL, Sorgel F (2007). Population pharmacokinetics and pharmacodynamics of continuous versus short-term infusion of imipenem-cilastatin in critically ill patients in a randomized, controlled trial. Antimicrob Agents Chemother.

[22] Ikawa K, Morikawa N, Uehara S, Monden K, Yamada Y, Honda N, Kumon H (2009). Pharmacokinetic-pharmacodynamic target attainment analysis of doripenem in infected patients. Int J Antimicrob Agents.

[23] Gumbo T, Louie A, Deziel MR, Liu W, Parsons LM, Salfinger M, Drusano GL (2007). Concentration-dependent Mycobacterium tuberculosis killing and prevention of resistance by rifampin. Antimicrob Agents Chemother.

[24] Rodvold KA, Nicolau DP, Lodise TP, Khashab M, Noel GJ, Kahn JB, Gotfried M, Murray SA, Nicholson S, Laohavaleeson S, Tessier PR, Drusano GL (2009). Identifying exposure targets for treatment of staphylococcal pneumonia with ceftobiprole. Antimicrob Agents Chemother.

[25] Kwa AL, Lim TP, Low JG, Hou J, Kurup A, Prince RA, Tam VH (2008). Pharmacokinetics of polymyxin B1 in patients with multidrug-resistant Gram-negative bacterial infections. Diagn Microbiol Infect Dis.

[26] National Committee for Clinical Laboratory Standards. Methods for Determining Bactericidal Activity of Antimicrobial Agents. Approved Guideline M26-A, Vol. 19. NCCLS. Wayne, PA;1999.

[27] Apisarnthanarak A, Li Yang H, Warren DK (2012). Termination of an extreme-drug resistant-acinetobacter baumannii outbreak in a hospital after flooding: lessons learned. Clin Infect Dis.

[28] Apisarnthanarak A, Pinitchai U, Warachan B, Warren DK, Khawcharoenporn T, Hayden MK (2014). Effectiveness of infection prevention measures featuring advanced source control and environmental cleaning to limit transmission of extremely-drug resistant Acinetobacter baumannii in a Thai intensive care unit: an analysis before and after extensive flooding. Am J Infect Control.

[29] Thapa B, Tribuddharat C, Srifuengfung S, Dhiraputra C (2010). High prevalence of bla(OXA)-23 in oligoclonal carbapenem-resistant Acinetobacter baumannii from Siriraj Hospital, Mahidol University, Bangkok, Thailand. Southeast Asian J Trop Med Public Health.

[30] Niumsup PR, Boonkerd N, Tansawai U, Tiloklurs M (2009). Carbapenem-resistant Acinetobacter baumannii producing OXA-23 in Thailand. Jpn J Infect Dis.

[31] Wang X, Qiao F, Yu R, Gao Y, Zong Z (2013). Clonal diversity of Acinetobacter baumannii clinical isolates revealed by a snapshot study. BMC microbiology.

[32] Lee G, Lee JH, Lim K, Suh IB, Ryu SW, Eom YB, Kim SH, Park M, Kim JB (2012). Prevalence of multidrug-resistant Acinetobacter baumannii producing OXA-23-like from a healthcare facility of Gangwon Province, South Korea. Int J Antimicrob Agents.

[33] Apisarnthanarak A, Mundy LM (2008). Role of combination antibiogram in empirical treatment of infection due to multidrug-resistant Acinetobacter baumannii. Infect Control Hosp Epidemiol.

[34] Hagihara M, Housman ST, Nicolau DP, Kuti JL (2013). In vitro pharmacodynamics of polymyxin B and tigecycline alone and in combination against carbapenem-resistant Acinetobacter baumannii. Antimicrob Agents Chemother.

[35] Zusman O, Avni T, Leibovici L, Adler A, Friberg L, Stergiopoulou T, Carmeli Y, Paul M (2013). Systematic review and meta-analysis of in vitro synergy of polymyxins and carbapenems. Antimicrob Agents Chemother.

[36] Aydemir H, Akduman D, Piskin N, Comert F, Horuz E, Terzi A, Kokturk F, Ornek T, Celebi G (2013). Colistin vs. the combination of colistin and rifampicin for the treatment of carbapenem-resistant Acinetobacter baumannii ventilator-associated pneumonia. Epidemiol Infect.

[37] Durante-Mangoni E, Signoriello G, Andini R, Mattei A, De Cristoforo M, Murino P, Bassetti M, Malacarne P, Petrosillo N, Galdieri N, Mocavero P, Corcione A, Viscoli C, Zarrilli R, Gallo C, Utili R (2013). Colistin and rifampicin compared with colistin alone for the treatment of serious infections due to extensively drug-resistant Acinetobacter baumannii: a multicenter, randomized clinical trial. Clin Infect Dis.

[38] Khawcharoenporn T, Pruetpongpun N, Tiamsak P, Rutchanawech S, Mundy LM, Apisarnthanarak A (2014). Colistin-based treatment for extensively drug-resistant Acinetobacter baumannii pneumonia. Int J Antimicrob Agents.

[39] Santimaleeworagun W, Wongpoowarak P, Chayakul P, Pattharachayakul S, Tansakul P, Garey KW (2011). Clinical outcomes of patients infected with carbapenem-resistant Acinetobacter baumannii treated with single or combination antibiotic therapy. J Med Assoc Thai.

[40] Batirel A, Balkan II, Karabay O, Agalar C, Akalin S, Alici O, Alp E, Altay FA, Altin N, Arslan F, Aslan T, Bekiroglu N, Cesur S, Celik AD, Dogan M, Durdu B, Duygu F, Engin A, Engin DO, Gonen I, Guclu E, Guven T, Hatipoglu CA, Hosoglu S, Karahocagil MK, Kilic AU, Ormen B, Ozdemir D, Ozer S, Oztoprak N, Sezak N, Turhan V, Turker N, Yilmaz H (2014). Comparison of colistin-carbapenem, colistin-sulbactam, and colistin plus other antibacterial agents for the treatment of extremely drug-resistant Acinetobacter baumannii bloodstream infections. Eur J Clin Microbiol Infect Dis.

[41] Aaron SD, Ferris W, Henry DA, Speert DP, Macdonald NE (2000). Multiple combination bactericidal antibiotic testing for patients with cystic fibrosis infected with Burkholderia cepacia. Am J Respir Crit Care Med.

[42] Lang BJ, Aaron SD, Ferris W, Hebert PC, MacDonald NE (2000). Multiple combination bactericidal antibiotic testing for patients with cystic fibrosis infected with multiresistant strains of Pseudomonas aeruginosa. Am J Respir Crit Care Med.

[43] Saginur R, Stdenis M, Ferris W, Aaron SD, Chan F, Lee C, Ramotar K (2006). Multiple combination bactericidal testing of staphylococcal biofilms from implant-associated infections. Antimicrob Agents Chemother.

[44] Haja Mydin H, Corris PA, Nicholson A, Perry JD, Meachery G, Marrs EC, Peart S, Fagan C, Lordan JL, Fisher AJ, Gould FK (2012). Targeted Antibiotic Prophylaxis for Lung Transplantation in Cystic Fibrosis Patients Colonised with Pseudomonas aeruginosa Using Multiple Combination Bactericidal Testing. J Transplant.

